# Kinin B_1_ and B_2_ receptors mediate cancer pain associated with both the tumor and oncology therapy using aromatase inhibitors

**DOI:** 10.1038/s41598-023-31535-6

**Published:** 2023-03-17

**Authors:** Indiara Brusco, Gabriela Becker, Tais Vidal Palma, Micheli Mainardi Pillat, Rahisa Scussel, Bethina Trevisol Steiner, Tuane Bazanella Sampaio, Daniel Mendes Pereira Ardisson-Araújo, Cinthia Melazzo de Andrade, Mauro Schneider Oliveira, Ricardo Andrez Machado-De-Avila, Sara Marchesan Oliveira

**Affiliations:** 1 Graduate Program in Biological Sciences: Biochemistry Toxicology, Department of Biochemistry and Molecular Biology, Federal University of Santa Maria, Av. Roraima 1000, Camobi, Santa Maria, RS 97105-900 Brazil; 2 Department of Microbiology and Parasitology, Federal University of Santa Maria, Santa Maria, RS Brazil; 3 Graduate Program in Health Sciences, University of Extreme South Catarinense, Criciuma, SC Brazil; 4 Graduate Program in Pharmacology, Department of Physiology and Pharmacology, Federal University of Santa Maria, Santa Maria, RS Brazil; 5 Department of Cell Biology, Institute of Biological Sciences, University of Brasilia, Brasilia, DF Brazil

**Keywords:** Breast cancer, Cancer models, Cancer therapy, Pain, Pharmacology, Receptor pharmacology

## Abstract

Pain caused by the tumor or aromatase inhibitors (AIs) is a disabling symptom in breast cancer survivors. Their mechanisms are unclear, but pro-algesic and inflammatory mediators seem to be involved. Kinins are endogenous algogenic mediators associated with various painful conditions via B_1_ and B_2_ receptor activation, including chemotherapy-induced pain and breast cancer proliferation. We investigate the involvement of the kinin B_1_ and B_2_ receptors in metastatic breast tumor (4T1 breast cancer cells)-caused pain and in aromatase inhibitors (anastrozole or letrozole) therapy-associated pain. A protocol associating the tumor and antineoplastic therapy was also performed. Kinin receptors’ role was investigated via pharmacological antagonism, receptors protein expression, and kinin levels. Mechanical and cold allodynia and muscle strength were evaluated. AIs and breast tumor increased kinin receptors expression, and tumor also increased kinin levels. AIs caused mechanical allodynia and reduced the muscle strength of mice. Kinin B_1_ (DALBk) and B_2_ (Icatibant) receptor antagonists attenuated these effects and reduced breast tumor-induced mechanical and cold allodynia. AIs or paclitaxel enhanced breast tumor-induced mechanical hypersensitivity, while DALBk and Icatibant prevented this increase. Antagonists did not interfere with paclitaxel's cytotoxic action in vitro. Thus, kinin B_1_ or B_2_ receptors can be a potential target for treating the pain caused by metastatic breast tumor and their antineoplastic therapy.

## Introduction

Breast cancer is the most frequently diagnosed cancer in women worldwide, reaching 24.7% of new cases in 2020, a year that for the first time surpassed lung cancer^1^. An increase of 47.5% in these cases is expected for 2040, according to the 2020 GLOBOCAN Cancer Tomorrow tool. Bone metastases are responsible for causing the most debilitating breast cancer pain once it usually does not cause excruciating pain in its native tissue^2–4^. Chronic pain occurs in 74% of breast cancer survivors, of which 84% present moderate-intensity pain from 1 to 3 days per week^5^. These pain symptoms are characterized by mechanical and cold allodynia, ongoing pain, paresthesia, and phantom sensations, which compromise the patients’ quality of life once they are often improperly treated^2,5,6^. Thus, it is essential to deepen the knowledge regarding cancer pain mechanisms to develop more appropriate analgesics^2,7^.

Cancer pain etiology includes direct tumor infiltration/expansion, tumoral metastasis, and tumor- or stromal cell-derived mediators, such as bradykinin, which can sensitize or activate nociceptors^2,8^. Cancer pain also results from diagnostic and surgical methods or as an adverse effect of anticancer therapy^2,4,6–10^. Paclitaxel, for example, is one of the main chemotherapeutic agents used in breast cancer therapy^11^. Nevertheless, it causes acute and neuropathic pain syndrome that limits its use^9,12–14^.

Aromatase inhibitors (AIs), indicated for estrogen receptor-positive breast cancer, also cause painful symptoms^10,11,15^. More than a third of patients that receive AIs report muscle and joint pain (34–50%) characterized by morning stiffness and pain in the hands, knees, hips, lower back, and shoulders^8,10,16–19^, in addition to neuropathic pain^20^. The most commonly prescribed AIs are anastrozole and letrozole^21^, often recommended for 5–10 years^18,22^. However, nearly 20% of patients discontinue or do not adhere to treatment due to painful symptoms^10,20^, thus compromising the success of anticancer therapy. Therefore, studies investigating therapeutic interventions for relieving these painful symptoms in patients^16^ without interfering with anticancer treatments are needed.

Compelling evidence showed that transient receptor potential ankyrin 1 (TRPA1) channels and protease-activated receptor 2 contribute to AIs-associated pain,^15,23^ but the mechanisms underlying this pain have not yet been fully clarified. These same studies suggest that pro-inflammatory and pro-algesic mediators may activate signalling pathways contributing to pain development caused by AIs^15,23^. Since kinins are potent endogenous algogenic peptides involved in inflammatory and pain processes via B_1_ and B_2_ receptors^24,25^, and these receptors can interact intracellularly with TRPA1^26,27^, they may be involved in AIs-induced pain. Kinin receptors are G protein-coupled whose activation stimulates phospholipase C and intracellular mediators’ formation, with increased intracellular calcium, thus exciting the cells where these receptors are expressed^25,28^. Nociceptive neurons associated with pain modulation in the periphery and spinal cord and non-neuronal cells that express kinin B_1_ and B_2_ receptors contribute to painful processes^28–35^. This explains the ability of kinins to produce pain directly when bound to their B_1_ and B_2_ receptors. Due to this distribution in peripheral and central structures involved in the nociceptive transmission, these receptors can mediate acute and systemic chronic pain conditions such as fibromyalgia, neuropathy, and others^31,36–40^, including pain caused by chemotherapy drugs such as paclitaxel^12,13,41^. These characteristics of kinin receptors are also suggestive of a possible contribution to the AIs-caused pain symptoms.

Kinins also regulate breast cancer progression by acting as cell proliferation agents^42–45^. Interestingly, breast cancer patients present elevated kinin levels^46^, the human breast expresses both kinin B_1_ and B_2_ receptors, and antagonists of these receptors can inhibit breast cancer cell proliferation^42,43,45^. Thus, we hypothesized that kinins are involved in breast cancer pain and that kinin receptor antagonists attenuate breast tumor proliferation while treating the pain caused by the tumor and anticancer therapy. Therefore, using a breast cancer pain model in mice, we evaluated the involvement of the kinin B_1_ and B_2_ receptors in pain induced by breast tumor at metastatic-stage, AIs, or breast tumor combined with AIs or paclitaxel.

## Results

### AIs cause mechanical allodynia and reduce muscle strength

**Figure 1:**
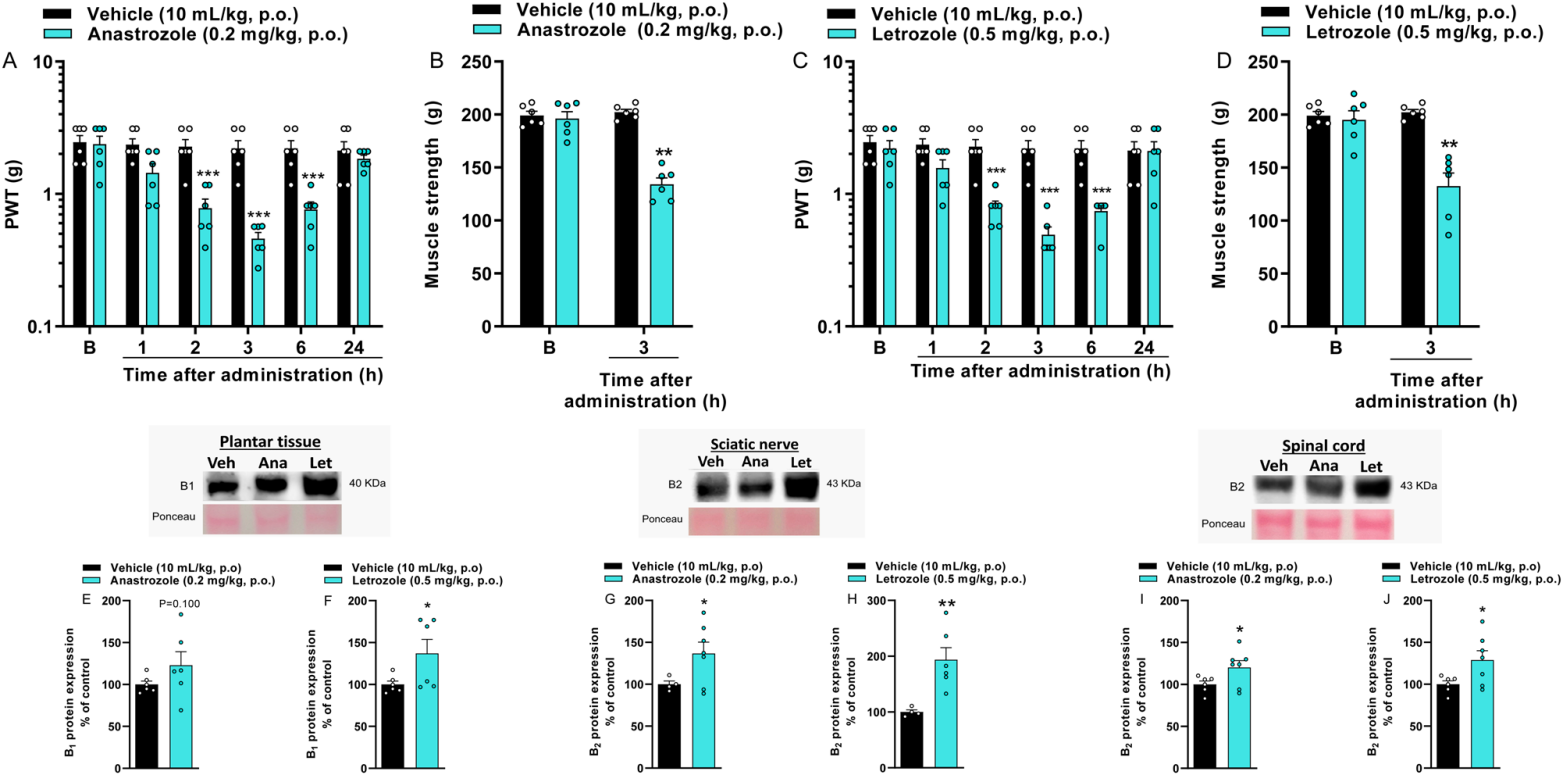
Aromatase inhibitors induce mechanical allodynia, reduce the muscle strength of mice and increase the kinin B_1_ and B_2_ receptor protein expression. Time-response curves to the mechanical allodynia (**A**,**C**) and the muscle strength at 3 h (**B**,**D**) after vehicle (10 mL/kg, p.o.), anastrozole (0.2 mg/kg, p.o. **A**,**B**), or letrozole (0.5 mg/kg, p.o. **C**,**D**) administration. (B) Denotes baseline mechanical threshold or muscle strength before drug administrations. Representative imagens of kinin B_1_ receptor protein expression in the plantar tissue (**E**,**F**), kinin B_2_ receptor protein expression in sciatic nerve (**G**,**H**) and spinal cord (**I**,**J**) at 3 h after vehicle (10 mL/kg, p.o.), anastrozole (0.2 mg/kg, p.o.) or letrozole (0.5 mg/kg, p.o.) administration. Data are expressed as the mean + SEM. *P < 0.05; **P < 0.01; ***P < 0.001 when compared to the vehicle group; two-way repeated-measures ANOVA followed by Bonferroni’s post hoc test to the behavioral tests (n = 6/group; C57BL/6 mice) or Student’s t-test to the protein expression (n = 4–7/group; C57BL/6 mice). *PWT* paw withdrawal threshold. The original western blot images are available in Supplementary Fig. S2.

AIs anastrozole (0.2 mg/kg, p.o.) and letrozole (0.5 mg/kg, p.o.) reduced the mechanical paw withdrawal threshold (PWT) of mice, indicating the development of mechanical allodynia. Mechanical allodynia was observed at 2, 3, and 6 h after anastrozole or letrozole administration with maximum PWT reduction of 79 ± 2% and 77 ± 3%, respectively, at 3 h after administrations ([F(5,50) = 11.85; P < 0.0001; Fig. 1A] and [F(5,50) = 9.58; P < 0.0001; Fig. 1C]). Anastrozole and letrozole also reduced the animals' muscle strength at 34 ± 3% and 35 ± 6%, respectively, at 3 h after its administrations (maximum nociception time observed in mechanical allodynia ([F(1,10) = 56.55; P < 0.0001; Fig. 1B] and [F(1,10) = 23.08; P < 0.001; Fig. 1D]).

### AIs increase kinin B_1_ and B_2_ receptor protein expression

The letrozole increased the kinin B_1_ receptor protein expression only in the plantar tissue, but anastrozole was not able to cause this increase (Fig. 1E,F). Both anastrozole and letrozole increased the kinin B_2_ receptor protein expression in the sciatic nerve and spinal cord (Fig. 1G–J) but not in plantar tissue (data not shown). However, the AIs did not alter bradykinin-related peptide levels in the plantar tissue (data not shown). Once AIs increased the kinin B_1_ and B_2_ receptors protein expression in peripheral and central structures of mice, despite limitations due to the lack of validity of antibodies specificity in knockout animals, we next evaluated the involvement of these receptors in nociceptive behaviors induced by AIs.

### Kinin B_1_ or B_2_ receptor antagonists relieve the AIs-induced mechanical allodynia and reduction on the muscle strength

**Figure 2:**
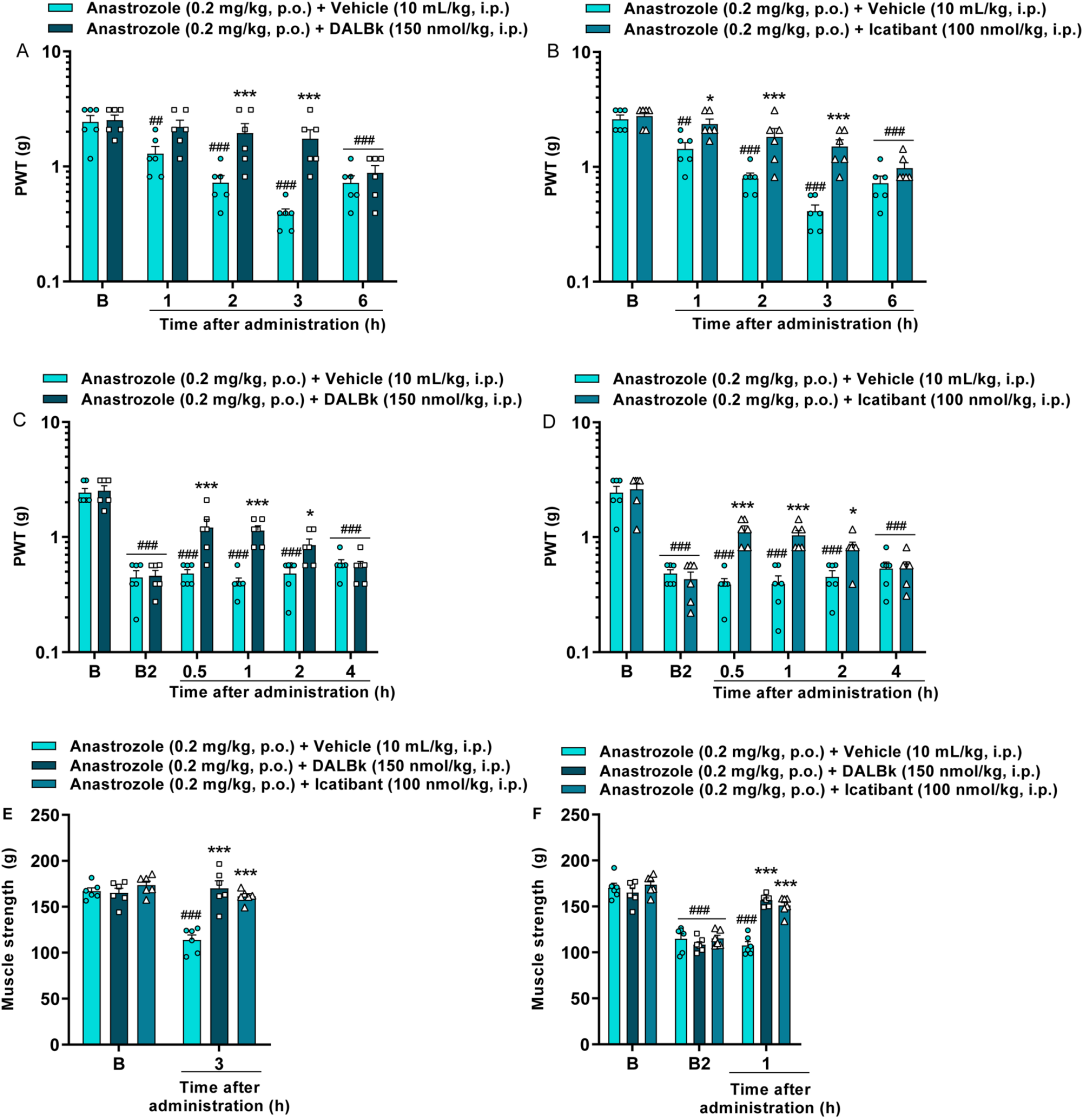
Kinin B_1_ (DALBk 150 nmol/kg, i.p.) and B_2_ (Icatibant 100 nmol/kg, i.p.) receptor antagonists relieve mechanical allodynia and reduction on the muscle strength caused by the aromatase inhibitor anastrozole (0.2 mg/kg, p.o.). Time-response curve for mechanical allodynia after treatment with DALBk or Icatibant injected 15 min before anastrozole administration (**A**,**B**). Time-response curve for mechanical allodynia after treatment with DALBk or Icatibant injected at 2 h after anastrozole administration (**C**,**D**). Measure muscle strength after treatment with DALBk or Icatibant injected 15 min before (**E**) or 2 h after (**F**) anastrozole administration. (B) Denotes baseline mechanical threshold or muscle strength before drug administration; B2 indicates baseline threshold at 2 h after anastrozole administration. Results are presented as mean + SEM (n = 6/group; C57BL/6 mice). ^##^P < 0.01; ^###^P < 0.001 compared to baseline threshold (B). *P < 0.05; ***P < 0.001 compared to the vehicle group. Two-way ANOVA repeated-measures followed by Bonferroni's post hoc test. *PWT* paw withdrawal threshold.

Pretreatment with the kinin B_1_ receptor antagonist DALBk reduced the anastrozole-induced mechanical allodynia at 2 and 3 h after administration with maximum inhibition (I_max_) of 66 ± 13% at 3 h [F(4,40) = 9.99; P < 0.0001; Fig. 2A]. The kinin B_2_ receptor antagonist Icatibant was effective from 1 to 3 h after administration with an I_max_ of 50 ± 8% in 3 h [F(4,40) = 9.67; P < 0.0001; Fig. 2B]. Post-treatment with DALBk and Icatibant also reduced anastrozole-induced mechanical allodynia from 0.5 to 2 h after administrations with I_max_ of 37 ± 7% and 33 ± 5% at 0.5 h, respectively ([F(5,50) = 8.72; P < 0.0001; Fig. 2C] and [F(5,50) = 11.05; P < 0.0001; Fig. 2D]). The reduction in muscle strength caused by anastrozole was prevented (DALBk inhibition = 100%; Icatibant inhibition = 90 ± 3%) and reversed (DALBk inhibition = 70 ± 4%; Icatibant inhibition = 61 ± 5%) by kinin B_1_ and B_2_ receptor antagonists ([F(2,15) = 23.25; P < 0.0001; Fig. 2E] and [F(4,30) = 20.31; P < 0.0001; Fig. 2F]).

**Figure 3:**
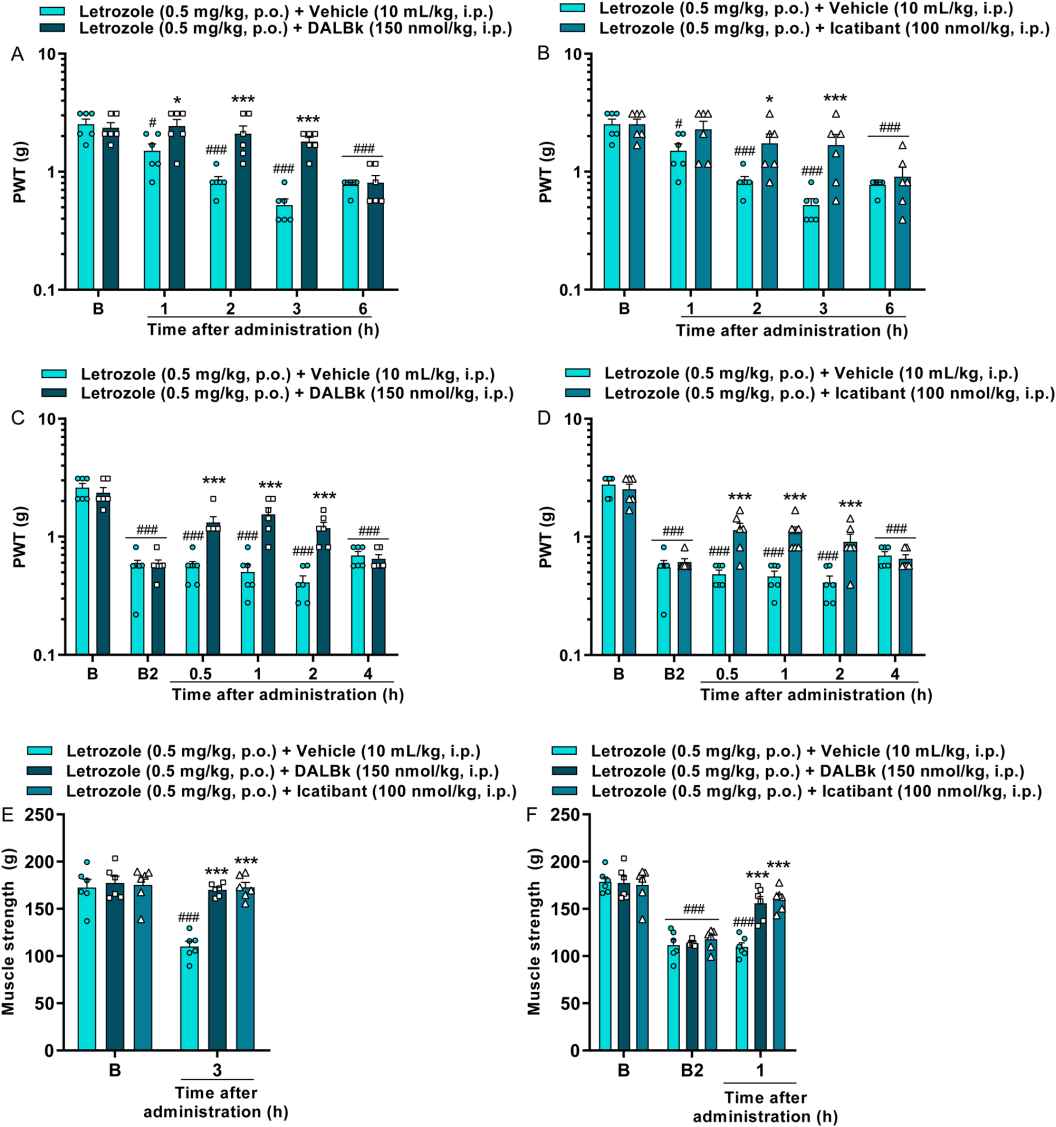
Kinin B_1_ (DALBk 150 nmol/kg, i.p.) and B_2_ (Icatibant 100 nmol/kg, i.p.) receptor antagonists relieve mechanical allodynia and reduction on the muscle strength caused by the aromatase inhibitor letrozole (0.5 mg/kg, p.o.). Time-response curve for mechanical allodynia after treatment with DALBk or Icatibant injected 15 min before letrozole administration (**A**,**B**). Time-response curve for mechanical allodynia after treatment with DALBk or Icatibant injected 2 h after letrozole administration (**C**,**D**). Measure muscle strength after treatment with DALBk or Icatibant injected 15 min before (**E**) or 2 h after (**F**) letrozole administration. (B) Denotes baseline mechanical threshold or muscle strength before drug administration while B2 indicates baseline threshold at 2 h after letrozole administration. Results are presented as mean + SEM (n = 6/group; C57BL/6 mice). ^#^P < 0.05; ^###^P < 0.001 compared to baseline threshold (B). *P < 0.05; ***P < 0.001 compared to the vehicle group. Two-way ANOVA repeated-measures followed by Bonferroni's post hoc test. *PWT* paw withdrawal threshold.

Similarly, pretreatment with DALBk reduced letrozole-induced mechanical allodynia from 1 to 3 h after administration with an I_max_ of 74 ± 16% in 2 h [F(4,40) = 11.89; P < 0.0001; Fig. 3A]. Icatibant was effective at 2 and 3 h after administration with an I_max_ of 58 ± 15% at 3 h [F(4,40) = 4.74; P < 0.01; Fig. 3B]. Post-treatment with DALBk and Icatibant also reduced letrozole-induced mechanical allodynia from 0.5 to 2 h after administrations with an I_max_ of 50 ± 8% at 1 h and 29 ± 5% at 0.5 h, respectively ([F(5,50) = 13.23; P < 0.0001; Fig. 3C] and [F(5,50) = 7.25; P < 0.0001; Fig. 3D]). The reduction in muscle strength caused by letrozole was also prevented (DALBk inhibition = 96 ± 3%; Icatibant inhibition = 100%) and reversed (DALBk inhibition = 76 ± 9%; Icatibant inhibition = 84 ± 6%) by kinin B_1_ and B_2_ receptor antagonists ([F(2,15) = 14.32; P < 0.01; Fig. 3E] and [F(4,30) = 8.97; P < 0.0001; Fig. 3F]).

### Breast tumor-bearing mice develop mechanical and cold allodynia

**Figure 4:**
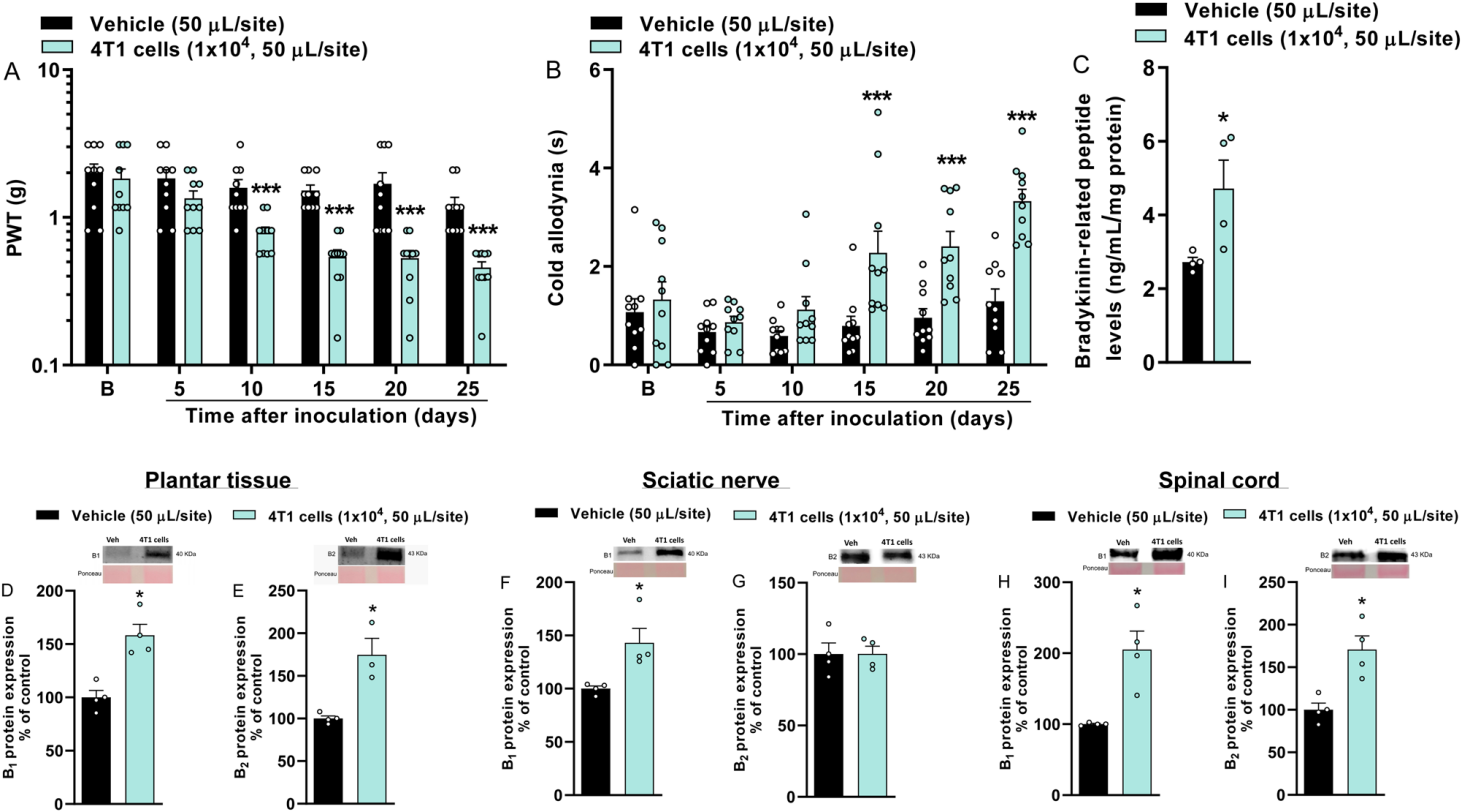
Metastatic breast tumor-bearing mice develop mechanical (**A**) and cold (**B**) allodynia and present increased bradykinin levels and kinin B_1_ and B_2_ receptor protein expression. The nociceptive parameters were evaluated at 5, 10, 15, 20, and 25 days after vehicle (50 µL/site) or 4T1 breast cancer cells (10^4^ cells, 50 µL/site) injection. (B) denotes baseline mechanical threshold/nociception time before injection. Bradykinin-related peptide levels in the plantar tissue (**C**). Representative images of kinin B_1_ and B_2_ receptor protein expression in the plantar tissue (**D**,**E**), in sciatic nerve (**F**,**G**), and spinal cord (**H**,**I**) at 20 days after vehicle (50 µL/site) or 4T1 breast cancer cells (1 × 10^4^, 50 mL/site) injection. Data are expressed as the mean + SEM. *P < 0.05; ***P < 0.001 when compared to the vehicle group; two-way repeated-measures ANOVA followed by Bonferroni’s post hoc test to the behavioral tests (n = 10/group; BALB/c mice) or Student’s t-test to the protein expression and kinin levels (n = 4/group; BALB/c mice). *PWT* paw withdrawal threshold. The original western blot images are available in Supplementary Fig. S3.

Metastatic breast tumor-bearing mice developed mechanical allodynia at 10, 15, 20, and 25 days after tumor induction with a maximum PWT reduction of 69 ± 4% at 20 days after injection [F(5,90) = 5.14; P < 0.01; Fig. 4A]. Cold allodynia was observed at 15, 20, and 25 days after tumor induction with greater nociception at 25 days [F(5,90) = 4.48; P < 0.01; Fig. 4B].

### Breast tumor-bearing mice present increased kinin B_1_ and B_2_ receptor protein expression and high bradykinin levels

The plantar tissue of metastatic breast tumor-bearing mice showed an increase in the bradykinin-related peptide levels of approximately twice (4.7 ± 0.8 ng/mL/mg protein) compared to the vehicle-injected mice (2.7 ± 0.2 ng/mL/mg protein) (Fig. 4C). Although there are limitations due to the lack of validity of the antibody specificity in kinin receptors knockout animals in our study, the protein expression of kinin B_1_ and B_2_ receptors increased in plantar tissue and spinal cord of breast tumor-bearing mice (Fig. 4D,E,H,I). Only the kinin B_1_ but not the B_2_ receptor protein expression increased in the sciatic nerve of these animals (Fig. 4F,G). Thus, we next evaluate the involvement of kinin receptors in metastatic breast cancer-associated nociceptive behaviors.

### Kinin B_1_ or B_2_ receptor antagonists relieve the breast tumor–induced mechanical and cold allodynia

**Figure 5:**
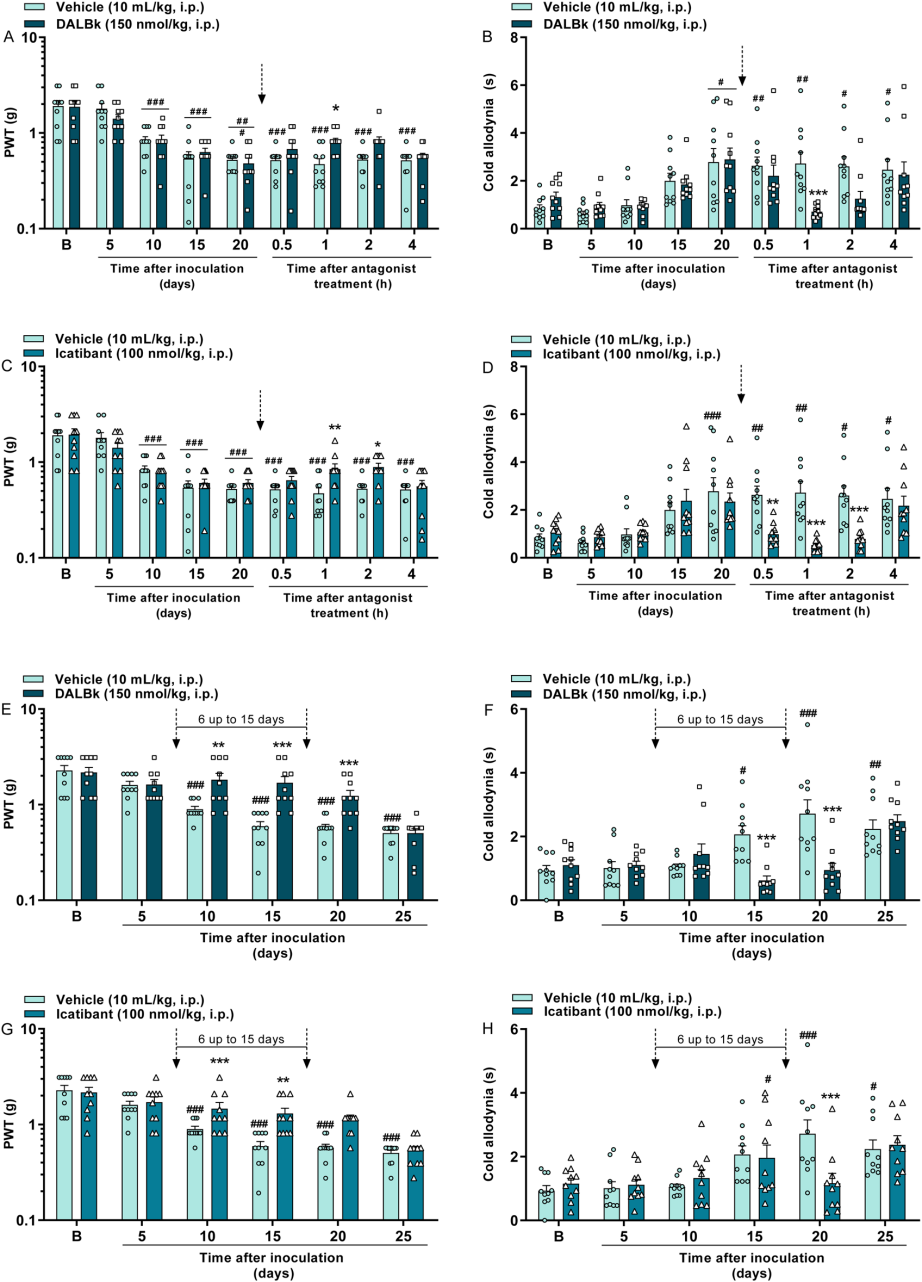
Kinin B_1_ (DALBk, 150 nmol/kg, i.p.) or B_2_ (Icatibant, 100 nmol/kg, i.p.) receptor antagonists reduce the mechanical (**A**,**C**,**E**,**G**) and cold (**B**,**D**,**F**,**H**) allodynia in breast tumor-bearing mice. Time-response curves caused by the post-treatment with the vehicle, DALBk (**A**,**B**) or Icatibant (**C**,**D**) at 20 days after 4T1 breast cancer cells (10^4^ cells, 50 µL/site) injection. Early-stage repeated treatment (6–15 days) with vehicle DALBk (**E**,**F**) or Icatibant (**G**,**H**) in animals that received 4T1 breast cancer cells (10^4^ cells, 50 µL/site) injection. (B) Denotes baseline mechanical threshold/nociception time before tumor induction. The dotted arrows indicate the timings of antagonist administration. Data are expressed as the mean + SEM (n = 10/group; BALB/c mice). ^#^P < 0.05; ^##^P < 0.01; ^###^P < 0.001 when compared to the baseline (B); *P < 0.05; ^**^P < 0.01; ***P < 0.001 when compared to the vehicle group; two-way repeated-measures ANOVA followed by Bonferroni’s post hoc test. *PWT* paw withdrawal threshold.

Kinin B_1_ (DALBk) and B_2_ (Icatibant) receptor antagonists caused an antinociceptive effect in mice 20 days after tumor induction, the day the maximum nociception was established. DALBk decreased the mechanical and cold allodynia induced by breast tumor at 1 h after its administration with inhibition of 24 ± 4% and 100%, respectively ([F(8,144) = 2.17; P < 0.05; Fig. 5A] and [F(8,144) = 2.97; P < 0.01; Fig. 5B]). Icatibant also decreased the mechanical allodynia at 1 and 2 h (I_max_ = 26 ± 7% at 1 h) and cold allodynia from 0.5 up to 2 h (inhibition = 100% at all times) after its administration ([F(8,144) = 2.22; P < 0.05; Fig. 5C] and [F(8,144) = 5.20; P < 0.0001; Fig. 5D]).

The repeated (from 6 up to 15 days after tumor injection) administration of kinin B_1_ and B_2_ receptor antagonists from the initial stage of tumor induction also promoted an antinociceptive effect in mice. DALBk decreased the mechanical and cold allodynia in breast tumor-bearing mice from 10 up to 20 days (I_max_ = 65 ± 10% at 15 days) and from 15 up to 20 days (inhibition = 100% at all times) after its administration, respectively ([F(5,90) = 7.99; P < 0.0001; Fig. 5E] and [F(5,90) = 9.01; P < 0.0001; Fig. 5F]). Likewise, Icatibant also decreased the mechanical allodynia in breast tumor-bearing mice at 10 and 15 days (I_max_ = 42 ± 11% at 15 days) and cold allodynia at 20 days (inhibition = 87 ± 15%) after its administration ([F(5,90) = 6.55; P < 0.0001; Fig. 5G] and [F(5,90) = 4.46; P < 0.01; Fig. 5H]).

### Anticancer drugs anastrozole, letrozole, and paclitaxel enhance the breast tumor-induced mechanical hypersensitivity

**Figure 6:**
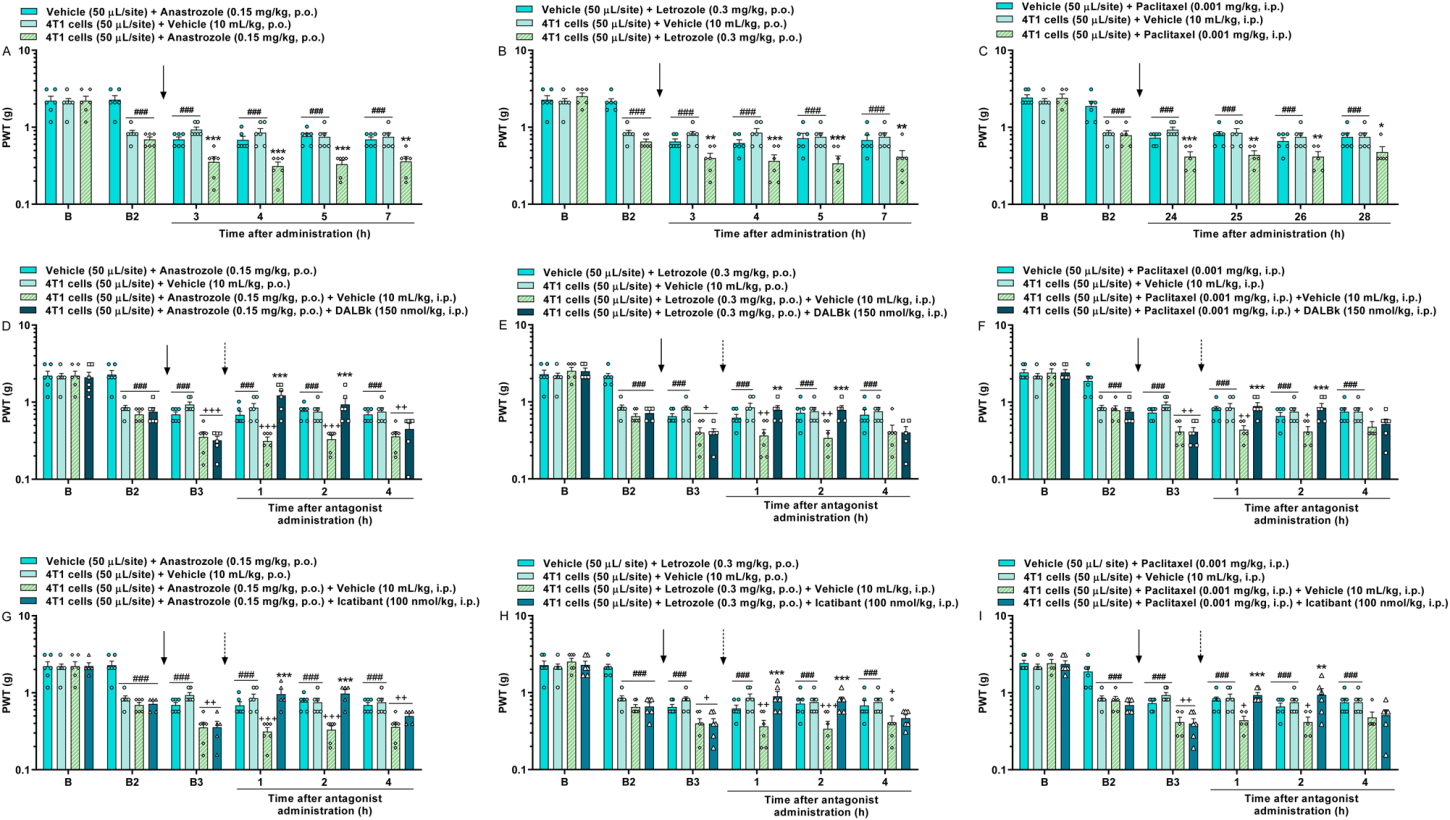
Anticancer drugs enhance the mechanical hypersensitivity in breast cancer-bearing mice, and kinin B_1_ (DALBk, 150 nmol/kg, i.p.) or B_2_ (Icatibant, 100 nmol/kg, i.p.) receptor antagonists reduce the mechanical hypersensitivity induced by breast tumor plus anticancer therapy. Time-response curves caused by the vehicle (10 mL/kg, p.o. or i.p.), anastrozole (0.15 mg/kg, p.o.; **A**), letrozole (0.3 mg/kg, p.o.; **B**) or paclitaxel (0.001 mg/kg, i.p.; **C**) administration at 10 days after vehicle (50 µL/site) or 4T1 breast cancer cells (10^4^ cells, 50 µL/site) injection. Time-response curves caused by the vehicle, DALBk (**D**–**F**) or Icatibant (**G**–**I**) treatment in animals previously injected with 4T1 breast cancer cells (10^4^ cells, 50 µL/site) plus anastrozole (0.15 mg/kg, p.o.; D;G), letrozole (0.3 mg/kg, p.o.;E;H) or paclitaxel (0.001 mg/kg, i.p.;F;I). (B) Denotes the baseline mechanical threshold before cells injection and drugs administration. (B2) denotes the baseline mechanical threshold at 10 days after cells injection and before drugs administration. (B3) denotes the baseline mechanical threshold at 10 days after cells injection and 3 h after anastrozole or letrozole administration, or 24 h after paclitaxel administration. The closed arrows indicate the timing of anticancer therapy administration, and the dotted arrows indicate timings of antagonist administration. Data are expressed as the mean + SEM (n = 5–6/group; BALB/c mice). ^###^P < 0.001 when compared to the baseline (B); ^+^P < 0.05 when compared to the 4T1 cells plus vehicle group; *P < 0.05; **P < 0.01; ***P < 0.001 when compared 4T1 cells plus anastrozole, letrozole or paclitaxel group; two-way repeated-measures ANOVA followed by Bonferroni’s post hoc test. *PWT* paw withdrawal threshold.

Breast cancer-bearing mice presented mechanical allodynia at 10 days (B2) after 4T1 breast cancer cells injection compared to the basal values (B). The administration of low doses of AIs anastrozole (0.15 mg/kg, p.o.) and letrozole (0.3 mg/kg, p.o.) enhanced the mechanical hypersensitivity induced by breast tumor from 3 up to 7 h after its administrations. The maximum PWT reduction was 63 ± 4% and 55 ± 10% at 4 h and 5 h after administration of anastrozole and letrozole, respectively ([F(10,75) = 5.61; P < 0.0001; Fig. 6A and [F(10,75) = 4.27; P < 0.001; Fig. 6B]). Likewise, low dose paclitaxel (0.001 mg/kg, i.p.) administration also enhanced the mechanical hypersensitivity in breast cancer-bearing mice from 24 to 28 h after its administration with a maximum PWT reduction of 55 ± 6% at 24 h [F(10,70) = 4.17; P < 0.001; Fig. 6C]. The cold hypersensitivity induced by breast tumor was not enhanced by the anticancer drugs (data not shown).

Although two animal strains were used in our study due to different pain models and histocompatibility of BALB/c mice with 4T1 breast cancer cells, the mechanical PWT reduction caused by anticancer drugs was reproduced in both mice strains. This is because BABL/c mice injected with the vehicle in the breast and treated with anastrozole and letrozole developed mechanical allodynia (Fig. 6A,B), as well as C57BL/6 mice treated with anastrozole and letrozole (Fig. 1A,C).

### Kinin B_1_ or B_2_ receptor antagonists reduce the mechanical hypersensitivity in a cancer pain model associated with the breast tumor and anticancer therapy

Low doses of AIs anastrozole (0.15 mg/kg, p.o.) and letrozole (0.3 mg/kg, p.o.) enhanced the breast tumor-induced mechanical hypersensitivity (B2) at 3 h after its administrations (B3). The treatment with kinin B_1_ (DALBk; 150 nmol/kg, i.p.) or B_2_ (Icatibant; 100 nmol/kg, i.p.) receptor antagonists reduced the mechanical hypersensitivity induced by breast tumor plus anastrozole at 1 and 2 h after antagonist administrations with I_max_ of 51 ± 9% for DALBk and 36 ± 7% for Icatibant at 1 h ([F(15,100) = 6.96; P < 0.0001; Fig. 6D] and [F(15,95) = 7.21; P < 0.0001; Fig. 6G]). DALBk and Icatibant also reduced the mechanical hypersensitivity induced by breast tumor plus letrozole at 1 and 2 h after its administrations with I_max_ of 25 ± 5% and 31 ± 7% at 2 h, respectively ([F(15,95) = 4.43; P < 0.0001; Fig. 6E] and [F(15,100) = 4.97; P < 0.0001; Fig. 6H]).

A low dose of paclitaxel (0.001 mg/kg, i.p.) also enhanced the breast tumor-induced mechanical hypersensitivity (B2) at 24 h after its administration (B3). The treatment with DALBk or Icatibant reduced the mechanical hypersensitivity induced by breast tumor plus paclitaxel at 1 and 2 h after its administrations with I_max_ of 27 ± 5% for DALBk and 31 ± 4% for Icatibant at 1 h ([F(15,95) = 5.19; P < 0.0001; Fig. 6F] and [F(15,95) = 4.60; P < 0.0001; Fig. 6I]).

Although the mechanical threshold has been partially restored to the baseline values (B) by kinin B_1_ (DALBk) or B_2_ (Icatibant) receptor antagonists, both DALBk and Icatibant reduced at 100% the potentiation of mechanical hypersensitivity induced by anastrozole, letrozole, or paclitaxel in breast cancer-bearing mice when compared to these mice treated with vehicle (Fig. 6).

### Expression of kinin B_1_ or B_2_ receptor in 4T1 breast cancer cells from Sequence Read Archive (SRA) analysis

We found reads mapped against the kinin B_1_ or B_2_ receptor coding DNA sequence (CDS) for the four independent transcriptome experiments (Supplementary Table S1). The PUM1 gene was used as an internal control and presented > 4900 mapped reads for each individual experiment. Kinin B_1_ receptor was transcribed in 4T1 cells at a residual level (only 28.5 ± 7.3 mapped reads) compared to the transcription of the kinin B_2_ receptor (1608.3 ± 46.3 mapped reads). Therefore, 4T1 cells grown in vitro seem to transcribe about 60-fold more kinin B_2_ receptor mRNA than the kinin B_1_ receptor. Once 4T1 cells appear to express kinin receptors, we next evaluated the effect of kinin receptor antagonists on these cells in vitro.

### Kinin B_1_ or B_2_ receptor antagonists did not alter the cytotoxic action of paclitaxel on 4T1 breast cancer cells

As expected, the chemotherapy paclitaxel (100 μM) reduced the 4T1 breast cancer cell viability by inhibiting 47 ± 3%. In the concentration tested, the kinin B_2_ receptor antagonist (Icatibant 100 μM) did not alter the 4T1 breast cancer cells viability. Interestingly, the kinin B_1_ receptor antagonist (DALBk, 100 μM) reduced the 4T1 breast cancer cells viability by inhibiting 40 ± 2%, similar to the paclitaxel. Relevantly, Icatibant (100 μM) or DALBk (100 μM) did not interfere with the cytotoxic action of paclitaxel (100 μM) when both were associated (Supplementary Fig. S1A).

Once DALBk, but not Icatibant, reduced the viability of 4T1 breast cancer cells in the MTT assay, we evaluated the effect of this antagonist in the apoptosis and cell migration assay. DALBk (100 μM) did not cause apoptosis of 4T1 cells in the annexin/7AAD assay (Supplementary Fig. S1B), nor did it reduce cell migration (Supplementary Fig. S1C). In contrast, paclitaxel (100 μM) caused early apoptosis in 40 ± 6% of cells and reduced cell migration by 83 ± 2% (Supplementary Fig. S1B,C). Notably, DALBk (100 μM) did not interfere with the effect of paclitaxel (100 μM) in inducing apoptosis (at 38 ± 5% of cells) and reducing migration (84 ± 4%) of 4T1 breast cancer cells when both were combined (Supplementary Fig. S1B,C).

## Discussion

Breast cancer patients experience chronic pain that may be associated with the tumor itself, especially after metastasis, and/or the anticancer therapy^2,4,5^. Cancer pain significantly affects the patient’s functional capacity, survival, and quality of life since the available treatments are partially effective or cause adverse effects^2,8,47^. Treatment guidelines from the World Health Organization (WHO) and other national or international bodies are broad and do not relate treatment approaches to pain classification^8^. This can occur because cellular and molecular mechanisms associated with cancer pain are still uncertain. In this sense, the kinins and their B_1_ and B_2_ receptors appear attractive targets to alleviate cancer-associated pain since they are involved in various painful conditions, including those induced by chemotherapy^12,13,39–41^ and contribute to tumor cell proliferation^42,43^. Here, our findings extend the use of kinin B_1_ and B_2_ receptor antagonists to treating the pain caused by the metastatic breast tumor, antineoplastic therapy with AIs, or both besides reducing breast tumor progression.

AIs are widely used as endocrine therapy for breast cancer^11^. However, breast cancer patients undergoing AIs treatment often report musculoskeletal symptoms, particularly arthralgias and myalgias characterized by pain and stiffness^11,16,17,19^. Here, the AIs anastrozole and letrozole elicited mechanical allodynia (pain from normally non-painful stimuli)^2,5^ and reduced the muscle strength of mice, in agreement with previous reports^15,23^.

In addition to TRPA1 channels, the contribution of pro-inflammatory and painful mediators in AIs-caused nociception was previously suggested^15^. Bradykinin and kallidin are endogenous peptides that mediate inflammatory and painful processes via the kinin B_2_ receptor, while their active metabolites (des-Arg-kinins) are kinin B_1_ receptor agonists^24,25^. Notably, activation of kinin receptors can lead to intracellular sensitization of TRPA1 through downstream vias (phospholipase C, protein kinase C, and protein kinase A), which can contribute to the pronociceptive effects of kinins^26,27^. Thus, AIs could be causing pain directly by activating kinin receptors and indirectly by sensitizing TRPA1, which is under investigation (Fialho et al., unpublished data). Here, we found that kinin B_1_ and B_2_ receptor antagonists attenuated the mechanical allodynia and loss of muscle strength caused by AIs. However, bradykinin-related peptide levels did not alter in the plantar tissue of mice after AIs administration.

Although the bradykinin content has not changed, both anastrozole and letrozole increased the kinin B_2_ receptor protein expression in the sciatic nerve and spinal cord but not plantar tissue, and letrozole increased the kinin B_1_ receptor protein expression in the plantar tissue of the mice. Kinin receptors are expressed in peripheral nociceptive neurons such as C- and Aδ-fibers, sensory ganglions, and the spinal cord, mediating nociceptive transmission^28–31^. Kinin receptors also are found or upregulated in astrocytes and microglia in the central nervous system, contributing to chronic pain and inflammatory states^32–35^. In response to nociceptive inputs, kinins also are released in the spinal cord, where they act in postsynaptic receptors potentiating glutamatergic synaptic transmission to produce pain hypersensitivity^48,49^. Additionally, the interaction of kinins with endothelial cells and leukocytes in peripheral tissues may contribute to conditions such as cancer, inflammation, and pain^31,50,51^. These data explain the ability of kinin B_1_ and B_2_ receptor antagonists to attenuate the AIs-induced mechanical allodynia and changes in muscle strength test, a sensitive test for painful parameters^52^. Assays of pharmacological antagonism, genetic manipulation, and receptor proteins expression showed kinins’ contribution to the pathogenesis of various acute and chronic pain models^31,36–40^, including chemotherapy-induced as vincristine^41^ and paclitaxel^12,13^. Paclitaxel is widely used in breast cancer management^11^, although it causes acute and neuropathic pain syndrome limiting its use. Notably, kinins contribute to developing both of these processes^9,12,13^. Thus, kinin receptor antagonists could advantage breast cancer patients by reducing the paclitaxel-induced pain syndrome and subsequently alleviating the pain associated with endocrine therapy with AIs.

Once kinins are involved in breast cancer cell proliferation^42,43^ and pain caused by antineoplastic therapy^13,15^, we assessed the role of kinin receptors in metastatic breast tumor-caused pain. The cancer pain model was induced by injecting 4T1 breast cancer cells into the mammary fat pads of female mice. This model mimics breast carcinoma's clinical signs and progression, inducing naturally bone metastases and nociception^53,54^, without the need for direct bone injection as performed on other models. Thus, the tumor progression is similar to human breast cancer^54,55^.

Hypersensitivity to touch and cold are clinical characteristics of cancer pain^56^. We found that metastatic breast tumor-bearing mice develop mechanical and cold allodynia as previously reported^3,54,57–59^. Most studies describe the development of breast cancer-induced bone pain^3,4,57,58,60^ and, in general, solid tumors, including mammary carcinoma, do not result in pain in their native tissue but cause excruciating pain once they metastasize to bone^3^. This observation reinforces the validity of this pain model in mimicking the clinical symptoms of breast cancer once 4T1 cells have a metastatic profile.

Tumor metastases cause bone remodeling and tissue injury, which presumably induces the release of bradykinin^2^. Additionally, tumor cells and the associated immune cells release algogenic substances, such as bradykinin, prostaglandins, and growth factors, which may be involved in the cancer pain^2,4,30,61^. Bradykinin, for example, was considered a potential target in developing analgesics for cancer pain treatment^2^. Here, metastatic breast tumor-bearing mice presented high kinin levels in the plantar tissue and a greater expression of kinin receptors protein in plantar tissue, sciatic nerve, and spinal cord than control mice. Interestingly, patients with breast cancer present higher serum levels of bradykinin and its metabolites compared to healthy individuals^46^. Similarly, kinin B_1_ receptor mRNA expression increased in mice's dorsal root ganglion in a melanoma-like skin cancer pain model^62^.

Kinin B_1_ and B_2_ receptor antagonists also reduced metastatic breast tumor-induced mechanical and cold allodynia. This effect was observed even during the maximal nociception stage (20 days after breast tumor induction). Further, antagonists seem more effective when administered in the early stages of tumor development. These findings corroborate previous studies in which pharmacologic blockade of the kinin B_1_ receptor attenuated pain-related behaviors during both early and advanced stages of bone cancer in mice^63^. The kinin B_1_ and B_2_ receptors also appear to be partially involved in nociceptive behaviors associated with melanoma-like skin cancer in mice^62^.

The tumor and the antineoplastic therapy were individually sufficient to trigger cancer pain. Additionally, anastrozole, letrozole, and paclitaxel enhanced the mechanical but not cold hypersensitivity caused by tumor cells in metastatic breast tumor-bearing animals. These results were already expected since AIs have been associated with the development of mechanical allodynia and changes in muscle strength, but not cold allodynia^15,23^. However, paclitaxel and the breast tumor cause mechanical and cold allodynia^13,54,59^. Still, the development of mechanical allodynia caused by the metastatic breast tumor occurred before (from day 10) the development of cold allodynia (from day 15), according to previous data^54^. Thus, the submaximal dose of paclitaxel was probably not enough to cause cold allodynia when both paclitaxel and the breast tumor were associated on day 10. Importantly, increased pain due to the use of anticancer therapies is correlated with a worse prognosis, suggesting that adequate analgesic interventions could improve the survival of cancer patients^64^.

Once it is difficult to define if the pain of cancer patients undergoing anticancer therapy is caused by the tumor or by the antineoplastic treatment, an analgesic capable of reducing the pain from both etiologies is clinically relevant. Here, kinin B_1_ and B_2_ receptor antagonists reduced the mechanical hypersensitivity in metastatic breast cancer-bearing mice, which is enhanced by anastrozole, letrozole, or paclitaxel. These results are relevant once kinin receptor antagonism reduces the pain symptoms caused by the tumor and by chemotherapy treatment with paclitaxel^13^, as well as by endocrine therapy with AIs, alone or associated with the breast tumor.

It is essential to observe whether analgesic drugs used to relieve cancer pain could interfere with tumor progression, impacting patient survival^2^. Although controversial, morphine’s long term use, for example, seems to impair survival and contribute to chemotherapy resistance and cell proliferation in breast cancer models in vitro and mice^66–68^. In this sense, kinin receptor antagonists seem advantageous since kinins exercise regulatory control over breast cancer progression by acting as cell proliferation agents and mediating pain. Consequently, kinin receptor antagonists exhibit antiproliferative and pro-apoptotic effects against breast carcinoma cells^42–45^. Nevertheless, when subjectively evaluated by visual observation and palpation, peptide kinin B_1_ and B_2_ receptor antagonists did not seem to interfere with tumor growth in our in vivo experiments. A larger 4T1 breast cancer cell concentration^69^, a more extended experimental period, and measurements that more accurately assess the tumor size and its metastases are necessary to observe tumor growth changes in vivo. However, it was previously discussed that at 30 days after cell injection, the advanced-stage tumor could reduce the animals' locomotor activity, thus compromising the detection of evaluated nociceptive parameters^54^. Thus, a careful experimental design is need to clarify these issues.

Although previous publications have established breast cancer models induced by 4T1 cells as metastatic^53,54^, a limitation of our study is the absence of these data since it is essential to confirm the tumor formation and its metastases to the bone and correlate them with pain behaviors. Besides breast tumors, it remains to be explored whether other metastatic tumors and different animal strains reproduce our data since our study was limited to BALB/c mice because this strain is histocompatible with 4T1 breast cancer cells. Another limitation of our study is the lack of confirmation of the specificity of the antibodies and data on the kinin receptors expression in a more specific manner in cells and tissues, such as the dorsal root ganglion or dorsal horn of the spinal cord, for example, which are essential in the processing of pain. Altogether, these investigations could better clarify our findings, especially the responsiveness to the pain of each animal evaluated.

Cell culture experiments showed that kinin B_1_ or B_2_ receptor peptide antagonists did not affect the cytotoxic action of paclitaxel on 4T1 breast cancer cells in the cell viability, migration, and apoptosis assays. Additionally, kinin B_1_ receptor peptide antagonist (DALBk), but not B_2_ (Icatibant), reduced 4T1 cell viability. Although SRA analysis showed that these cells present both kinin receptors, the B_2_ receptor is 60 times more expressed. Perhaps a higher concentration of its antagonist would be necessary for experiments in these cells. Furthermore, DALBk alone was ineffective against cell migration and apoptosis. The peptide characteristic of these antagonists may have influenced this lack of antiproliferative effect since kinin receptor peptide antagonists cannot cross cell membranes and, thus, may fail to produce anticancer effects. However, non-peptide antagonists and cell-permeable peptide antagonists have shown potential value against breast cancer proliferation and cooperate with suboptimal doses of chemotherapy (doxorubicin and paclitaxel) to promote anticancer effects^42,43^. Notably, kinin receptor antagonists appear more cytotoxic to cancer cells than normal breast cells, an important aspect of new anticancer therapies^43^. The breast cancer cells express various members of the kallikrein-kinin system, supporting the hypothesis that kinins may be formed in the tumor microenvironment and autoregulate functionality of tumor cells^70^. Therefore, the role of kinin receptor antagonists in breast cancer proliferation should be further explored in human cells and tissue samples.

The pain associated with the tumor and its therapy is often treated as a distinct entity from cancer itself. However, it must be an integral part of the treatment to improve patients’ quality of life and survival^2,47,64^. Analgesics recommended to treat cancer pain, such as opioids and non-steroidal anti-inflammatory drugs, do not always lead to complete pain relief and may also cause various adverse effects^47^. Consequently, new cellular and molecular mechanism-based therapies are necessary to reduce cancer-associated pain^2,7^. Our findings show the involvement of the kinin B_1_ and B_2_ receptors in pain caused by tumor and antineoplastic therapy with AIs. Since cancer pain is a mixed type of inflammatory and neuropathic pain^8^, future investigations into the relationship of kinins at specific sites in the spinal cord and with peripheric inflammatory components may further clarify their role in breast cancer pain associated with metastatic-stage tumor and AIs. Importantly, Icatibant, a kinin B_2_ receptor antagonist approved for hereditary angioedema, is described as safe and well tolerated by patients^71^. Therefore, kinin B_1_ or B_2_ receptors present a potential target in relieving the pain associated with metastatic breast cancer and its therapy without compromising the effect of antineoplastic agents while still contributing to disease progression control.

## Methods

### Materials

Anastrozole and letrozole were purchased from Tocris Bioscience (Bristol, UK) and were diluted in 0.5% carboxymethylcellulose and 99.5% of NaCl (0.9%). Paclitaxel (6 mg/mL of paclitaxel in Cremophor EL and dehydrated ethanol) was purchased from Glenmark (Buenos Aires, ARG) and was dissolved in NaCl (0.9%). Icatibant (kinin B_2_ receptor peptide antagonist), des-Arg^9^-[Leu^8^]-bradykinin (DALBk; kinin B_1_ receptor peptide antagonist) were purchased from Sigma-Aldrich Chemical Company (St. Louis MO, USA) and were also prepared in NaCl (0.9%). The enzyme immunoassay kit for bradykinin was obtained from Phoenix Pharmaceuticals, Inc. (California, USA). Specific anti-B_1_ (bs-8675R–lot 9C20V14) or anti-B_2_ (bs-2422R–lot AG08307921) antibodies were acquired from Bioss Antibodies (Massachusetts, USA) and secondary antibody (sc-2357–lot L1218) was obtained from Santa Cruz Biotechnology (California, USA). Paclitaxel from semisynthetic ≥ 97% (in vitro assays) was acquired from Sigma-Aldrich Chemical Company (St. Louis MO, USA). Annexin-V and 3- (4, 5-dimethyl-2-thiazolyl) -2, 5-diphenyl-2H-tetrazolium bromide] salt (MTT; 5 mg/mL solution) were obtained from Life Technologies (São Paulo, BR). The 7-Amino-Actinomycin D (7AAD) was acquired from BD Biosciences (California, USA). The doses of the drugs used in this study were based on previous studies^13,15,23,40,72^.

### Animals

Experiments were conducted using female C57BL/6 mice (20–25 g) and female BALB/c mice (20–30 g), which were maintained in a temperature-controlled room (22 ± 1 °C) under a 12 h light/12 h dark cycle with free access to food and water. Behavioral assessments were conducted between 8:00 a.m. and 5:00 p.m. C57BL/6 mice were used in the pain protocols induced by AIs. In contrast, BALB/c mice were used in the pain protocols caused by tumor alone, tumor plus AIs, and tumor plus paclitaxel once 4T1 cells have histocompatibility with BALB/c mice. All protocols were approved by the Institutional Animal Care and Use Committee of the Federal University of Santa Maria (process #4647180719/2019 for C57BL/6 mice) or by the Institutional Animal Care and Use Committee of the University of Extreme South Catarinense (process #71/2019-1-UNESC for BALC/c mice). Experimental protocols followed ethical guidelines established for investigations of experimental pain in conscious animals^73^. The experiments also were performed following the national and international legislation (guidelines of Brazilian Council of Animal Experimentation Control—CONCEA—and of U.S. Public Health Service’s Policy on Humane Care and Use of Laboratory Animals—PHS Policy) and the Animal Research: Reporting in vivo Experiments (ARRIVE) guidelines^74^. The number of animals and the intensities of noxious stimuli used were the minimum necessary to demonstrate the consistent effects of the treatments. Animals were allocated according to the baseline thresholds before and after the drugs administration or cells injection, according to the experimental protocol. All experiments were also performed by experimenters blinded to drug administration or the group to be tested.

### Mechanical allodynia

The mechanical allodynia was evaluated with von Frey filaments of increasing stiffness (0.02–10 g) using the Up-and-Down method^75,76^. The mechanical paw withdrawal threshold (PWT) was calculated according to Dixon (1980)^77^ and expressed in grams (g). The mechanical allodynia was considered a decrease in the PWT compared to the baseline (B) values (before induction of the pain models).

### Cold allodynia

The cold allodynia was assessed through the nocifensive response to the acetone-evoked evaporative cooling^78^. A droplet (20 µL) of acetone was gently applied to the plantar surface of the animals’ hind paws, and the time spent in elevation and licking of the plantar region was measured for 60 s. Cold allodynia was considered as an increase in the nociceptive response caused by exposure to acetone compared with basal (B) values (before induction of the pain models).

### Muscle strength—grip test

The muscle strength of mice was measured by an automated grip strength meter (Model EFF305, Insight, São Paulo, Brazil). The apparatus consists of a raised metal grid from the floor and is connected to a power transducer. Mice were placed on the grid and allowed to grip it with their paws, and then they were gently pulled backward in a horizontal plane from the tail base. The maximum strength exerted by the paws of each mouse was automatically recorded in grams by the device, and results were expressed as muscle strength in grams (g). The test was repeated three times per mouse with at least 1 min resting period between each test^79^.

### AIs-induced pain model

For the induction of the pain model, the mice received the oral administration (p.o.) via gavage of the AIs anastrozole (0.2 mg/kg, p.o) or letrozole (0.5 mg/kg, p.o.). A group of animals received only the oral vehicle administration (0.5% carboxymethylcellulose in saline (0.9% NaCl))^15^.

### Evaluation of the mechanical allodynia and muscle strength after AIs administration

The mechanical PWT and muscle strength were measured before (baseline values; B) or after vehicle (10 mL/kg, p.o.), anastrozole (0.2 mg/kg, p.o) or letrozole (0.5 mg/kg, p.o.) treatment^15^.

### Effect of kinin B_1_ or B_2_ receptor antagonists on AIs-induced mechanical allodynia and reduction of the muscle strength

The effect of kinin receptor antagonists on AIs-induced mechanical allodynia and reduced muscle strength was evaluated in a pre-treatment and post-treatment protocol. For the pre-treatment protocol, the mechanical PWT and muscle strength of animals were measured (baseline values; B). After that, animals were intraperitoneally treated with vehicle (10 mL/kg, i.p.), or kinin B_1_ (DALBk, 150 nmol/kg, i.p.), or B_2_ (Icatibant, 100 nmol/kg, i.p.) receptor antagonists. After 15 min, the animals received anastrozole (0.2 mg/kg, p.o.) or letrozole (0.5 mg/kg, p.o.), and the mechanical PWT was evaluated at 1, 2, 3, and 6 h after AIs administration. The muscle strength was measured at 3 h after AIs administration.

For the post-treatment protocol, the baseline mechanical threshold and muscle strength (baseline values; B) were assessed before anastrozole (0.2 mg/kg, p.o.) or letrozole (0.5 mg/kg, p.o.) treatments and at 2 h after its administrations (baseline values B2). The animals presenting mechanical allodynia and reduced muscle strength were treated with vehicle (10 mL/kg, i.p.), or kinin B_1_ (DALBk, 150 nmol/kg, i.p.), or B_2_ (Icatibant, 100 nmol/kg, i.p.) receptor antagonists. Mechanical PWT was evaluated at 0.5, 1, 2, and 4 h after antagonists’ administrations, while the muscle strength was measured at 1 h after the antagonist’s administrations.

### Breast tumor-induced pain model

The metastatic breast cancer pain model associated with the tumor was induced by injecting 4T1 murine breast carcinoma cells as previously determined^54^. The 4T1 cells (ATCC® CRL2539TM®) were obtained from Banco de Células do Rio de Janeiro, Brazil (code 0022) and were free of mycoplasma contamination. The 4T1 cells were cultured using DMEM medium in monolayer supplemented with 5% fetal bovine serum and 1% penicillin/streptomycin. For tumor induction, the cells were resuspended in PBS, and 50 μL of the cell suspension (10^4^ cells) or vehicle (PBS) was injected into the right fourth caudal mammary fat pad of female mice.

### Evaluation of metastatic breast tumor-induced mechanical and cold allodynia

Mechanical PWT and cold sensitivity were measured before (baseline values; B) the injection of the vehicle or 4T1 breast cancer cells. Next, animals received vehicle (50 µL/site) or 4T1 breast cancer cells (10^4^ cells, 50 µL/site) in the right fourth mammary gland, and mechanical PWT and cold sensitivity were again assessed at 5, 10, 15, 20 and 25 days after injection^54^.

### Effect of kinin B_1_ or B_2_ receptor antagonists on breast tumor-induced mechanical and cold allodynia

A reversion protocol was performed to assess whether kinin receptor antagonists could reduce mechanical and cold allodynia during a stage of maximum nociception induced by breast tumor. The mechanical PWT and cold sensitivity were measured before (baseline values; B) and several days (5, 10, 15, and 20) after injection of 4T1 breast cancer cells (10^4^ cells, 50 µL/site). At 20 days after cells injection (day of maximum nociception observed in previous tests), animals received intraperitoneally (i.p.) vehicle (10 mL/kg, i.p.) or kinin B_1_ (DALBk; 150 nmol/kg, i.p.) or B_2_ (Icatibant; 100 nmol/kg, i.p.) receptor antagonists, and the mechanical PWT and cold sensitivity were again evaluated from 0.5 up to 4 h after treatments.

Another protocol was carried out to assess whether treatment with kinin receptor antagonists during an early stage could reduce the mechanical and cold allodynia induced by breast tumor. Mechanical PWT and cold sensitivity were measured before (baseline values; B) and 5 days after the injection of 4T1 breast cancer cells (10^4^ cells, 50 µL/site) to show that nociception was not yet established. Next, animals received vehicle (10 mL/kg, i.p.) or kinin B_1_ (DALBk; 150 nmol/kg, i.p.) or B_2_ (Icatibant; 100 nmol/kg, i.p.) receptor antagonists from 6 up to 15 days after tumor injection, totaling ten administrations. The mechanical PWT and cold sensitivity were re-evaluated at 10, 15, 20, and 25 days after inoculation of the tumor, always after treatment with antagonists.

### Cancer pain model associated with the breast tumor and anticancer therapy

To assess whether treatment with AIs and the chemotherapy paclitaxel could potentiate the pain caused by the breast tumor, we performed a cancer pain protocol associated with the tumor and anticancer therapy. The mechanical PWT was measured before (baseline values; B) and at 10 days (baseline values; B2) after the vehicle (50 µL/site) or 4T1 breast cancer cells (10^4^ cells, 50 µL/site) injection. Next, the animals received vehicle (10 mL/kg, p.o.), low doses of anastrozole (0.15 mg/kg, p.o.) or letrozole (0.3 mg/kg, p.o.), or a low dose of paclitaxel (0.001 mg/kg, i.p.). The mechanical PWT was again evaluated at 3, 4, 5, and 7 h after anastrozole or letrozole administration; or at 24, 25, 26, and 28 h after paclitaxel administration.

Once the mechanical PWT was partially reduced at 10 days after the tumor cells injection, this time was selected to observe the mechanical hypersensitivity potentiation effects with anticancer drugs. For the same reason, low doses of anticancer drugs were used in this protocol according to previous studies^13,23^.

### Effect of kinin B_1_ or B_2_ receptor antagonists on the potentiation of mechanical hypersensitivity caused by breast tumor combined with anticancer therapy

The mechanical PWT was measured before (baseline values; B) and at 10 days (baseline values; B2) after the vehicle (50 µL/site) or 4T1 breast cancer cells (10^4^ cells, 50 µL/site) injection. The animals then received vehicle (10 mL/kg, p.o.) or low doses of anastrozole (0.15 mg, p.o.), letrozole (0.3 mg/kg, p.o.) or paclitaxel (0.001 mg/kg, i.p.). The mechanical PWT was evaluated at 3 h after anastrozole or letrozole administration or 24 h after paclitaxel administration corresponding to the third baseline value (B3). Next, animals were treated with vehicle (10 mL/kg, i.p.) or kinin B_1_ (DALBk; 150 nmol/kg, i.p.) or B_2_ (Icatibant; 100 nmol/kg, i.p.) receptor antagonists, and the mechanical PWT was again assessed from 1 up to 4 h after antagonist administrations.

### Determination of bradykinin-related peptide levels

The plantar tissue of the mice was collected at 3 h after vehicle (10 mL/kg, p.o.), anastrozole (0.2 mg/kg, p.o.), or letrozole (0.5 mg/kg, p.o.) administrations, and at 20 days after vehicle (50 µL/site) or 4T1 breast cancer cells (10^4^ cells, 50 µL/site) injection. Samples were homogenized in a buffer containing kininase inhibitors. The kinin levels were measured by enzyme immunoassay using a high-sensitivity kit for bradykinin. The results were expressed as bradykinin-related peptide levels in ng/mL of the sample^40,80^, normalized for mg/protein^81^.

### Protein expression of kinin B_1_ and B_2_ receptors

The plantar tissue, sciatic nerve, and spinal cord (T1-L6 approximately) of the mice were collected at 3 h after vehicle (10 mL/kg, p.o.), anastrozole (0.2 mg/kg, p.o.), or letrozole (0.5 mg/kg, p.o.) administrations, and at 20 days after vehicle (50 µL/site) or 4T1 breast cancer cells (10^4^ cells, 50 µL/site) injection. Briefly, samples were homogenized in a lysis buffer containing protease and phosphatase inhibitors. Protein content was determined using bovine serum albumin as the standard by the bicinchoninic acid method (Thermo Fisher Scientific, Massachusetts, USA). Proteins were submitted to SDS-PAGE (8% resolving gels) and transferred to nitrocellulose membranes. The membranes were blocked with 3% bovine serum albumin and incubated overnight at 4 °C with rabbit anti-B_1_ (1:1000) and anti-B_2_ (1:1000) polyclonal primary antibodies and for 1 h at room temperature with anti-rabbit secondary antibody (1:5000). The chemiluminescence was developed with ECL solution in a ChemiDoc™ MP Image System (Bio-Rad, California, USA)^40^. The blots were cut before hybridization to save the primary antibodies, approximately between 60 and 30 kDa (ColorBurst™ Electrophoresis Marker, # C1992, Sigma Aldrich). Also, due to unspecific bands, some blots were covered to the adequate obtention of protein immunoreactivity. Protein immunoreactivity was measured with ImageJ software. Ponceau S was used as a loading control. Each value was expressed as the ratio between arbitrary units obtained by the kinin B_1_ or B_2_ receptors bands and the respective ponceau area^82^. The results are shown as protein expression % related vehicle-treated controls.

### Sequence read archive (SRA) experiment analysis

Through Sequence Read Archives (SRAs) available at the NCBI platform, we search for kinin B_1_ and B_2_ receptor transcripts in deep-sequencing 4T1 breast cancer cells grown in vitro. Bioproject PRJNA533191 contains the transcriptomes of 4T1 cells. Briefly, total RNAs from 4T1 cells were extracted in quadruplicate; four barcoded mRNA-seq cDNA libraries were individually prepared and deep-sequenced using the Illumina HiSeq 2000 platform. The transcriptome reads were downloaded using the SRA Toolkit, trimmed, and individually mapped against the entire coding DNA sequence (CDS) of kinin B_1_ receptor (NM_007539), kinin B_2_ receptor (NM_009747), and as internal control PUM1 (AY027917) of mice using the Geneious R9.0 Software^83^. The reads were mapped to the sequence with a pairwise nucleotide identity of 99%.

### Cell viability measurement

The possible cytotoxic effect of the kinin B_1_ or B_2_ receptor antagonists was evaluated through MTT assay^84^. The 4T1 breast cancer cells were cultured overnight in a 96-well plate (2 × 10^5^ cells/mL) in a culture medium at 37 °C and 5% CO_2_. Next, the medium was discarded, and cells were treated with vehicle/control (only DMEM medium), kinin B_1_ (DALBk; 100 μM) or B_2_ (Icatibant; 100 μM) receptor antagonists, or paclitaxel (100 μM) and incubated at 37 °C and 5% CO_2_ for 72 h. In another set of experiments, cells were incubated for 72 h with DALBk (100 μM) or Icatibant (100 μM) associated with the paclitaxel (100 μM) to observe if the kinin B_1_ or B_2_ receptor antagonists interfere with the chemotherapeutic action of paclitaxel. The in vitro protocol was performed with paclitaxel since it has direct cytotoxic action, unlike AIs considered an adjuvant endocrine treatment for breast cancer.

The drugs' concentrations were defined using a previously performed concentration–response curve (1, 10, and 100 μM). After the incubation period, the medium was discarded, and 40 μL of MTT solution was added to each well. One hour later, the MTT was removed, and 120 μL of dimethylsulfoxide was added to solubilize the formazan salts. The experiments were repeated in triplicate. The results were colorimetrically determined at 570 nm and expressed as the cell viability percentage compared with the control.

### Cell apoptosis and migration assay

Once DALBk (kinin B_1_ receptor antagonist) reduced the viability of 4T1 breast cancer cells in the MTT assay, we also tested this antagonist in the cell apoptosis and migration assay. For apoptosis assay, 4T1 breast cancer cells were cultured in 6-well plates (2 × 10^5^ cells/well) in the presence of vehicle/control (only DMEM medium), DALBk (100 µM), paclitaxel (100 µM), or DALBk (100 µM) combined with paclitaxel (100 µM) for 48 h. After the treatment period, the supernatant was discarded, and the cells were removed from the wells with a trypsin/EDTA solution (0.2%/0.02%). The trypsin activity was neutralized with the addition of a medium with 10% fetal bovine serum. Subsequently, cells were centrifuged, supernatants were discarded, and cell pellets were resuspended with 200 μL of annexin V binding buffer (10 mM HEPES, 140 mM NaCl, 2.5 mM CaCl_2_, and 1 μL annexin-V) plus 7AAD (5 μL). The samples were incubated at room temperature for 30 min and immediately acquired in a BD FACSCalibur flow cytometer. Data were analyzed with Flowjo V10 software and expressed as the percentage of cells in early apoptosis.

In the cell migration assay, the 4T1 breast cancer cells were incubated in 24-well plates with DMEM medium supplemented with 10% fetal calf serum until confluency. Then, a scratch was made with a sterile tip in the central region of the well for mechanical removal of the cells. The treatment with DALBk (100 µM), paclitaxel (100 µM), or DALBk (100 µM) combined with paclitaxel (100 µM) was added for 24 h^85^. Cell migration capacity was assessed by comparing images of the wells before and after treatments using ImageJ software (NIH, USA). The results were expressed as the percentage of cell migration concerning the control.

### Statistical analyses

Statistical analyses were performed using GraphPad Prism 6.0 software. Results were expressed as the mean and standard error of the mean (SEM). The significance of differences between groups was evaluated with a Student’s *t*-test, one-way or two-way (time and treatment as factors; F values indicate the interaction between these factors) analysis of variance (ANOVA) followed by Bonferroni’s post hoc test. Mechanical threshold data were transformed to log before analyses to attend to parametric assumptions. P values lower than 0.05 (P < 0.05) were considered significant.

## Supplementary Information

Supplementary Information.

## Data Availability

Data sources and handling of the publicly available datasets used in this study are described in the Methods section. Further details and other data supporting the findings of this study are available from the corresponding authors upon request.
