# Supplementary material for: Kinin B1 and B2 receptors mediate cancer pain associated with both the tumor and oncology therapy using aromatase inhibitors
Source: Sci Rep. 2023 Mar 17;13:4418. doi: 10.1038/s41598-023-31535-6 (PMC10023805; doi:10.1038/s41598-023-31535-6)
Supplement: Supplementary file 1 — Supplementary Information. [file 41598_2023_31535_MOESM1_ESM.docx]

**Kinin B_1_ and B_2_ receptors mediate breast cancer pain associated with both the tumor and oncology therapy using aromatase inhibitors**

Indiara Brusco^1*^_,_ Gabriela Becker^1^, Tais Vidal Palma^1^_,_ Micheli Mainardi Pillat^2,^ Rahisa Scussel^3^, Bethina Trevisol Steiner^3^, Tuane Bazanella Sampaio^4^, Daniel Mendes Pereira Ardisson-Araújo^1^, Cinthia Melazzo de Andrade^1^, Mauro Schneider Oliveira^4^, Ricardo Andrez Machado-De-Avila^3^, Sara Marchesan Oliveira^1*^ ORCID: 0000-0003-2960-5284

**Results**

**Expression of kinin B_1_ or B_2_ receptor in 4T1 breast cancer cells from Sequence Read Archive (SRA) analysis**

| **Supplementary Table S1**. Kinin B_1_, B_2_ receptor, and Pum1-mapped reads individually recovered from the 4T1 cell transcriptome SRA experiments. | | | | |
| --- | --- | --- | --- | --- |
| **SRAs** | **Spots** | **Number of mapped reads** | | |
|  |  | **B_1_** | **B_2_** | **PUM1** |
| **SRX5692211** | 79,791,262 | 25 | 1,574 | 4,939 |
| **SRX5692212** | 78,172,738 | 20 | 1,629 | 4,789 |
| **SRX5692213** | 86,470,592 | 33 | 1,665 | >5,000 |
| **SRX5692214** | 78,727,044 | 36 | 1,567 | >5,000 |
|  | **Average** | 28.5 | 1,608.75 | 4,932 |
|  | **SD** | 7,3 | 46,6 | 99,6 |

**Kinin B_1_ or B_2_ receptor antagonists did not alter the cytotoxic action of paclitaxel on 4T1 breast cancer cells**


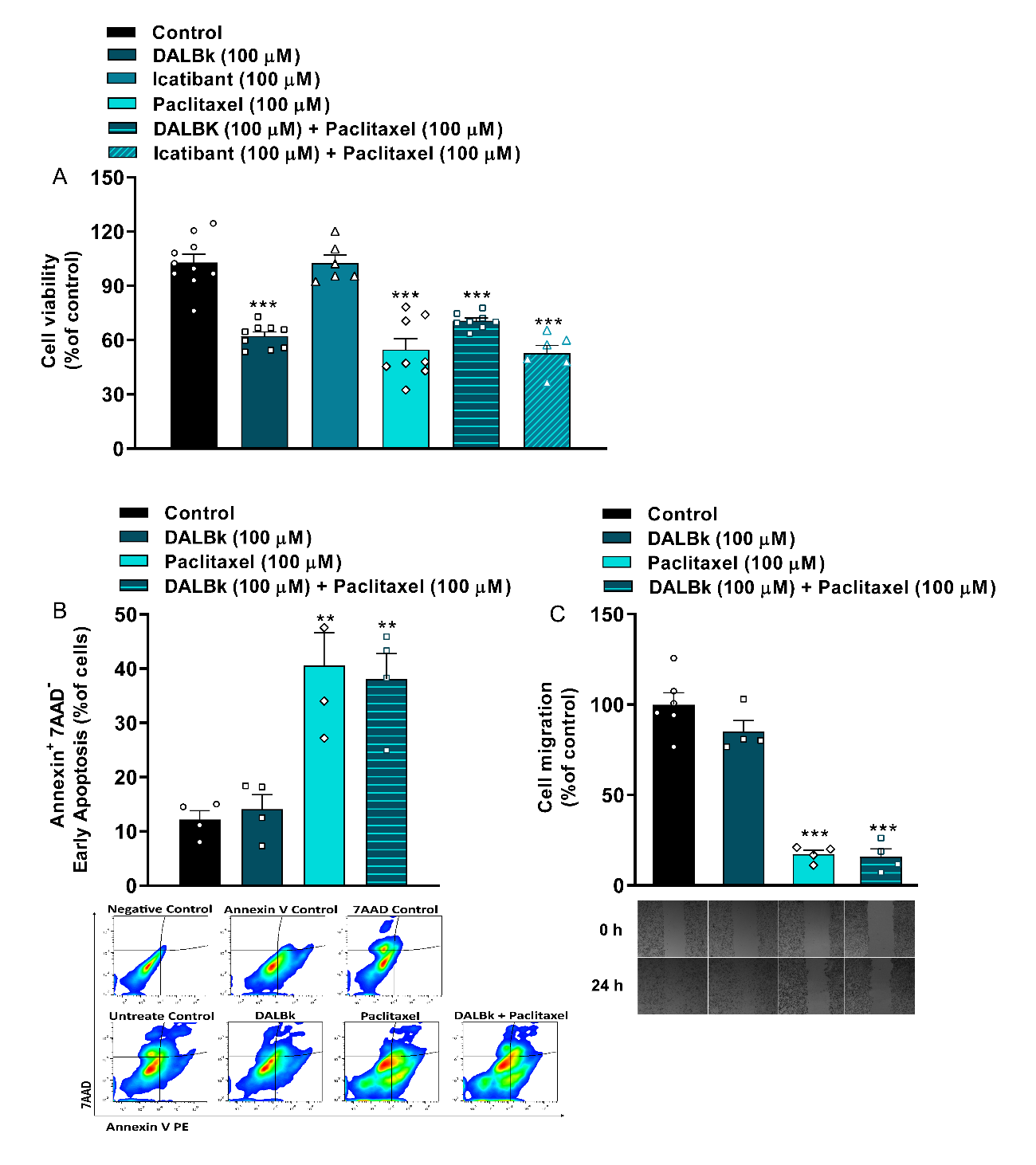


**Supplementary Fig. S1** Effect of the kinin B_1_ (DALBk) or B_2_ (Icatibant) receptor antagonists and of the paclitaxel on the 4T1 breast cancer cells (2x10^5^ cells/mL). (A) cell viability performed in triplicate, (B) apoptosis, and (C) migration assays. The control group denotes only DMEM culture medium (vehicle) (n=3-5 to A and n= 3-6 to B and C). **P<0.01; ***P<0.001 compared to the control (one-way ANOVA followed by Bonferroni’s post hoc test).


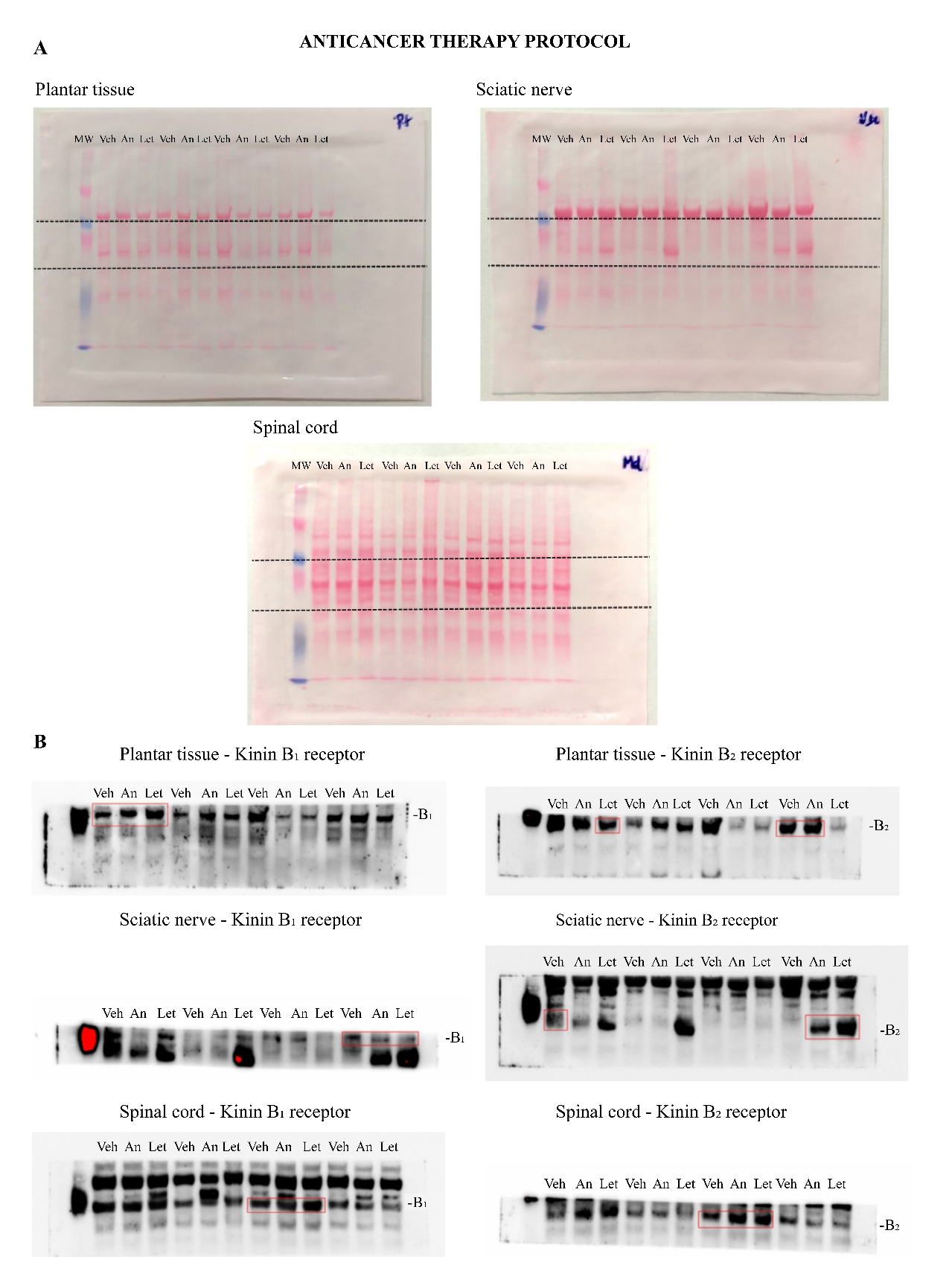


**Supplementary Fig. S2.** Original western blot images of kinin B_1_ and B_2_ receptor protein expression in plantar tissue, sciatic nerve, and spinal cord at 3 hours after vehicle (10 mL/kg, p.o.), anastrozole (0.2 mg/kg, p.o.) or letrozole (0.5 mg/kg, p.o.) administration. (A) Full-length membranes stained ponceau whose dashed lines indicate the region where the membrane was cut to hybridization with the antibodies. (B) The blots were cut before hybridization to save the primary antibodies, approximately between 60 kDa and 30 kDa (ColorBurst™ Electrophoresis Marker, # C1992, Sigma Aldrich). In addition, due to unspecific bands, some blots were covered to the adequate obtention of protein immunoreactivity. Red boxes denote the western blot bands used in the representative images.


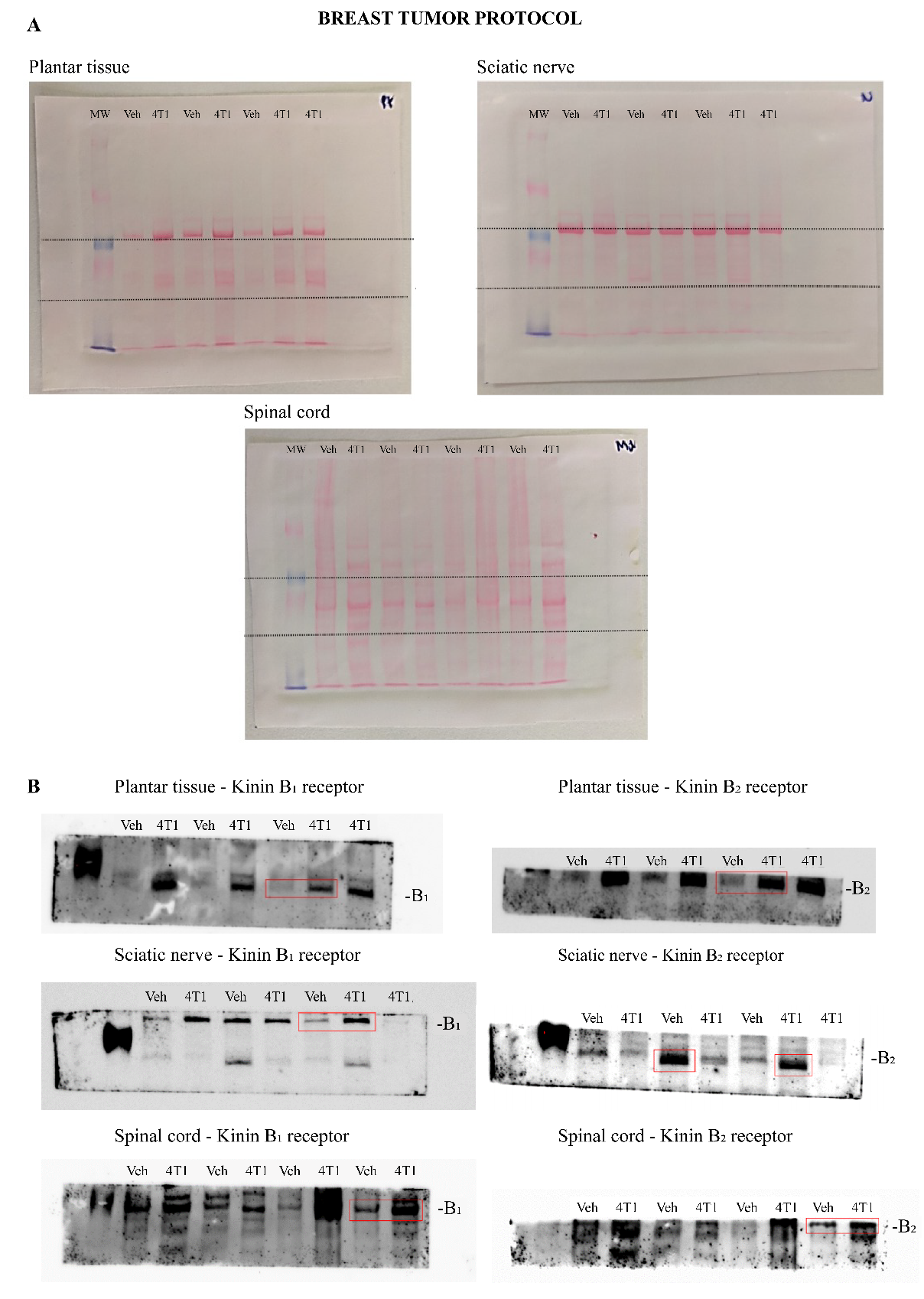


**Supplementary Fig. S3.** Original western blot images of kinin B_1_ and B_2_ receptor protein expression in plantar tissue, sciatic nerve, and spinal cord at 20 days after injection of vehicle (50 µL/site) or 4T1 breast cancer cells (1x10^4^, 50 µl/site). (A) Full-length membranes stained ponceau whose dashed lines indicate the region where the membrane was cut to hybridization with the antibodies. (B) The blots were cut before hybridization to save the primary antibodies, approximately between 60 kDa and 30 kDa (ColorBurst™ Electrophoresis Marker, # C1992, Sigma Aldrich). In addition, due to unspecific bands, some blots were covered to the adequate obtention of protein immunoreactivity. Red boxes denote the western blot bands used in the representative images.
